# Efficacy of Duloxetine in the Management of Diabetic Neuropathy: A Prospective Observational Cohort Study

**DOI:** 10.7759/cureus.82382

**Published:** 2025-04-16

**Authors:** Muhammad Osama, Maha Javaid, Bilal Shahid, Heema, Ayesha Ghazal Jamali, Bushra Anwar, Taimour Mushtaq

**Affiliations:** 1 Hematology, Singleton Hospital, Swansea, GBR; 2 Internal Medicine, Mayo Hospital, Lahore, PAK; 3 Psychiatry, Balochistan Institute of Psychiatry and Behavioral Science, Quetta, PAK; 4 Internal Medicine, Civil Hospital, Karachi, PAK; 5 Medicine, Liaquat University of Medical and Health Sciences, Jamshoro, PAK; 6 Endocrinology, Frontier Medical and Dental College, Abbottabad, PAK; 7 Medicine, Lahore Medical and Dental College, Lahore, PAK

**Keywords:** diabetic neuropathy, duloxetine, glycemic control, nerve conduction studies, neuropathic pain, visual analog scale (vas)

## Abstract

Background

Diabetic neuropathy (DN) is a prevalent complication of diabetes mellitus (DM), often leading to chronic pain and impaired quality of life. Duloxetine, a serotonin-norepinephrine reuptake inhibitor (SNRI), has been widely studied for its potential role in alleviating neuropathic pain.

Objective

This study aimed to evaluate the efficacy and safety of duloxetine in the management of DN.

Methods

A cross-sectional study was conducted in the Medicine Department of Sandeman Provincial Hospital, Quetta, from November 2022 to October 2023. A total of 113 patients aged between 25 and 65 years with clinically diagnosed DN were included. Patients received duloxetine, starting at 20 mg daily, with gradual dose escalation based on tolerance. Pain severity was assessed using the Visual Analog Scale (VAS), and nerve conduction studies (NCS) were performed at baseline, four weeks, and eight weeks post-treatment. Biochemical parameters, including fasting blood glucose (FBG), postprandial blood glucose (PPBS), and HbA1c levels, were measured at baseline. Adverse effects were recorded at each follow-up visit. Statistical analyses were conducted using IBM SPSS Statistics for Windows, Version 26.0 (Released 2019; IBM Corp., Armonk, NY, United States), with paired t-tests for continuous variables and chi-square tests for categorical variables. A p < 0.05 was considered statistically significant.

Results

The mean age of participants was 52.7 ± 8.1 years, with a mean diabetes duration of 6.3 ± 2.1 years. Eighty patients (70.8%) had poor glycemic control, with an HbA1c level > 6.5%. The mean baseline pain score on the VAS was 7.6 ± 1.1, which significantly reduced to 4.3 ± 0.9 at four weeks and 2.5 ± 0.8 at eight weeks (p < 0.001). NCS showed significant improvements in median and peroneal nerve conduction velocity, with mean median nerve conduction velocity increasing from 48.5 ± 6.2 m/s at baseline to 51.1 ± 5.9 m/s at four weeks and 54.3 ± 5.8 m/s at eight weeks (p < 0.001). Peroneal nerve conduction velocity also improved from baseline to follow-up at four weeks and eight weeks (p < 0.001). Adverse effects were reported in 32 (28.3%) patients, with nausea (12, 10.6%), dizziness (8, 7.1%), and somnolence (7, 6.2%) being the most common.

Conclusion

Duloxetine demonstrated significant efficacy in reducing neuropathic pain and improving nerve conduction parameters in patients with DN over an eight-week period. The drug was well tolerated, with mild and manageable adverse effects. These findings support the use of duloxetine as an effective and safe therapeutic option for managing DN.

## Introduction

Diabetes mellitus (DM) is a global health concern, affecting approximately 463 million adults worldwide, with an expected increase to 700 million by 2045 [1,2]. In 2000, there were an estimated 5.2 million adults in Pakistan who had diabetes, and by 2021, it increased to over 33 million. According to WHO estimates, 58% of Pakistan's total fatalities in 2016 were attributable to noncommunicable diseases (NCDs). In 2016, diabetes, cancer, heart disease, and chronic respiratory conditions were the four most common NCDs in Pakistan. According to a 2016 survey, 3% of all deaths were thought to be directly related to diabetes [3].

Among the various complications of diabetes, diabetic neuropathy (DN) is one of the most prevalent and debilitating conditions, affecting nearly 50% of individuals with long-standing diabetes [4]. DN leads to sensory, motor, and autonomic dysfunctions, with diabetic peripheral neuropathy (DPN) being the most common form [5]. Patients with painful DPN (PDPN) experience chronic neuropathic pain, significantly impairing their sleep, mood, and overall quality of life [6].

The pathophysiology of DN is multifactorial, involving hyperglycemia-induced oxidative stress, mitochondrial dysfunction, and neuroinflammation, ultimately leading to peripheral nerve damage [7]. Despite extensive research, there is no disease-modifying treatment for PDPN, and management remains symptomatic [6]. Current treatment strategies focus on pain relief, with pharmacological options including tricyclic antidepressants, anticonvulsants, opioids, and serotonin-norepinephrine reuptake inhibitors (SNRIs) [8].

Among the available therapies, duloxetine, an SNRI, has shown significant efficacy in PDPN [9]. It works by inhibiting the reuptake of serotonin and norepinephrine, modulating descending pain pathways in the central nervous system [10]. Clinical trials have demonstrated that duloxetine significantly reduces neuropathic pain, with a study by Wu et al. [9] reporting pain reduction scores greater than 50% over 12 weeks of therapy. Additionally, duloxetine has been associated with improved sleep patterns and overall patient well-being [11]. However, its efficacy may vary among populations. Despite its proven efficacy, limited local studies have evaluated duloxetine’s role in managing DN in Pakistan. Given the high burden of DN and its associated complications, it is crucial to explore effective treatment options that improve patients’ functional outcomes and quality of life.

Given the rising prevalence of diabetes and its complications, it is crucial to explore effective treatment options for PDPN. This study aims to evaluate the efficacy of duloxetine in the management of DN in a local population, addressing gaps in existing research. By identifying effective pharmacological interventions, this research could help reduce morbidity and improve patient quality of life in individuals suffering from DN.

## Materials and methods

This prospective observational cohort study was conducted in the Medicine Department of Sandeman Provincial Hospital, Quetta, from November 2022 to October 2023. The study aimed to evaluate the efficacy of duloxetine in the management of DN in patients with DM. It was approved by the Research Evaluation Unit, College of Physicians and Surgeons Pakistan, under reference number CPSP/REU/MED/2020/001/16418.

The sample size was determined using an anticipated proportion of 25% effect size, with an 8% margin of error and a 95% confidence interval, yielding a total of 113 participants. A non-probability, consecutive sampling technique was used to recruit 120 eligible patients to overcome the dropouts.

Inclusion and exclusion criteria

Patients aged between 25 and 65 years, with a confirmed diagnosis of DN and no prior treatment with any drug for neuropathy management, were included in the study. Neuropathic symptoms, including pain, numbness, tingling, or burning sensations in the extremities, were confirmed using the Michigan Neuropathy Screening Instrument (MNSI).

Patients younger than 25 or older than 65 years, those who were pregnant, or those who had taken duloxetine in the last three months were excluded. Patients with benign prostatic hyperplasia, prostate cancer, ischemic heart disease, arrhythmias, or other cardiac conditions were also excluded. Additionally, individuals with neuropathy due to other medical conditions such as hypothyroidism, leprosy, porphyria, or chronic renal failure were excluded based on clinical history and biochemical evidence.

Treatment and follow-up

All eligible patients were enrolled after obtaining written informed consent. Duloxetine therapy was initiated at 20 mg once daily at bedtime, with the dose gradually escalated to 20 mg each week, based on tolerability, up to a maximum dose of 120 mg per day. The mean dose achieved among patients was 94.5 ± 18.2 mg/day. Patients were evaluated at four and eight weeks for treatment response and adverse effects, and dosing adjustments were made accordingly to optimize therapeutic benefits while minimizing side effects. The final assessment of efficacy was conducted at 12 weeks.

Assessment parameters

Pain Severity Measurement

Pain severity was assessed using the VAS at baseline, four weeks, and eight weeks. A 50% reduction in pain score was considered a clinically significant response.

Neuropathy Assessment

Neuropathy symptoms and signs were evaluated using the MNSI at baseline and eight weeks post-treatment.

Nerve Conduction Studies (NCS)

Electrophysiological assessments were conducted at baseline, four weeks, and eight weeks using the Computerized RMS EMG EP MK II machine. Motor NCS were performed for the median, ulnar, common peroneal, and tibial nerves, measuring distal latency, amplitude, and conduction velocity. Sensory NCS were conducted for the median, ulnar, and sural nerves, evaluating sensory latency, amplitude, and conduction velocity. Standardized electrode placement and stimulation protocols were followed to ensure accuracy.

Anthropometric and Biochemical Measurements

Body weight, height, and body mass index (BMI) were recorded at baseline and eight weeks post-treatment. Body weight was measured using a digital weighing scale (±100 g accuracy), with patients wearing light clothing and no shoes. Height was measured to the nearest 0.5 cm, and BMI was calculated as weight (kg)/height² (m²).

Biochemical analyses included fasting blood glucose (FBG) and postprandial blood glucose (PPBG), measured using the oxidase-peroxidase method with a bichromatic autoanalyzer. Fasting samples were collected after 14 hours of fasting (between 08:00 and 09:00 AM), while postprandial samples were collected two hours after a meal. Glycated hemoglobin (HbA1c) levels were measured using the fast ion exchange resin separation method with a glycohemoglobin HbA1c test kit (human), analyzed through colorimetry.

Adverse Effect Monitoring

Adverse effects, including nausea, dizziness, somnolence, constipation, increased appetite, anorexia, dry mouth, and sweating, were recorded at each follow-up visit.

Statistical analysis

Data were analyzed using IBM SPSS Statistics for Windows, Version 26.0 (Released 2019; IBM Corp., Armonk, NY, United States). Continuous variables (e.g., pain scores, nerve conduction parameters, and biochemical measures) were expressed as mean ± standard deviation (SD) and analyzed using the repeated measures ANOVA for comparisons between baseline, four weeks, and eight weeks. A p < 0.05 was considered statistically significant. Categorical variables (e.g., adverse effects and gender distribution) were reported as n (%).

## Results

Demographic and baseline characteristics

The majority of patients were middle-aged and had long-standing diabetes, which is a significant risk factor for the development of DN. The mean age of participants was 52.7 ± 8.1 years, with the majority belonging to the 35-55 age group (77, 68.1%), while 22 (19.5%) were between 56 and 65 years. The mean duration of diabetes was 6.3 ± 2.1 years, with 65 (57.5%) having diabetes for ≤5 years and 48 (42.5%) for >5 years. The mean duration of neuropathic symptoms was 8.1 ± 3.5 months, with 47 (41.6%) experiencing symptoms for ≤6 months and 66 (58.4%) for >6 months (Table 1).

**Table 1:** Baseline characteristics of the study participants Data are presented as mean ± SD and N (%).

Characteristic	N (%)	Mean ± SD
Age (years)		52.7 ± 8.1
25-34	14 (12.4%)	
35-55	77 (68.1%)	
56-65	22 (19.5%)	
Duration of diabetes (years)		6.3 ± 2.1
≤5	65 (57.5%)	
>5	48 (42.5%)	
Duration of neuropathy (months)		8.1 ± 3.5
≤6	47 (41.6%)	
>6	66 (58.4%)	
BMI (kg/m²)		26.4 ± 3.2
Normal (18.5-24.9)	35 (31.0%)	
Overweight (25-29.9)	57 (50.4%)	
Obese (≥30)	21 (18.6%)	

The mean BMI at baseline was 26.4 ± 3.2 kg/m², with 35 (31.0%) classified as normal weight (18.5-24.9 kg/m²), 57 (50.4%) as overweight (25-29.9 kg/m²), and 21 (18.6%) as obese (≥30 kg/m²).

Glycemic control and biochemical parameters

The findings indicate that a majority of the patients had poor glycemic control, which could contribute to neuropathy progression and severity. The mean HbA1c at baseline was 7.8 ± 1.3%, with 80 (70.8%) having levels greater than 6.5%, indicating poor glycemic control. The mean FBG was 176.4 ± 21.5 mg/dL, and the mean PPBS was 224.6 ± 28.1 mg/dL (Table 2).

**Table 2:** Glycemic control and biochemical parameters Data presented as mean ± SD and N (%).

Parameter	N (%)	Mean ± SD	Reference range
HbA1c ≤ 6.5 (%)	33 (29.2%)		<6.5%
HbA1c > 6.5 (%)	80 (70.8%)	7.8 ± 1.3	
Fasting blood glucose (mg/dL)		176.4 ± 21.5	<126 mg/dL
Postprandial blood glucose (mg/dL)		224.6 ± 28.1	<200 mg/dL

Pain severity and neuropathy symptoms (Visual Analog Scale and Michigan Neuropathy Screening Instrument scores)

Pain severity, measured using the VAS, showed a significant reduction from baseline to four and eight weeks post-treatment. The mean baseline pain score was 7.6 ± 1.1, which significantly reduced to 4.3 ± 0.9 at four weeks and 2.5 ± 0.8 at eight weeks (p < 0.001) (Table 3).

**Table 3:** Changes in pain scores (VAS) and neuropathy symptoms (MNSI) after duloxetine treatment Data presented as mean ± SD. Repeated measures ANOVA applied; p < 0.05 is considered statistically significant.

Time point	VAS score (mean ± SD)	MNSI score (mean ± SD)	F-value	p-value
Baseline	7.6 ± 1.1	6.4 ± 1.3		
Four weeks	4.3 ± 0.9	5.2 ± 1.1	58.2	<0.001
Eight weeks	2.5 ± 0.8	4.1 ± 1.1	77.9	<0.001

Neuropathy severity, assessed using the MNSI, also showed improvement. The mean MNSI score decreased from 6.4 ± 1.3 at baseline to 5.2 ± 1.1 at four weeks and 4.1 ± 1.1 at eight weeks (p < 0.001), indicating a notable reduction in neuropathic symptoms (Table 3).

Nerve conduction studies

NCS demonstrated statistically significant improvements in key parameters of the median and peroneal nerves following treatment at four and eight weeks (p < 0.001). Specifically, median nerve latency decreased from 4.2 ± 0.6 ms at baseline to 3.9 ± 0.5 ms at four weeks and further to 3.5 ± 0.5 ms at eight weeks. Similarly, peroneal nerve latency improved from 5.1 ± 0.8 ms to 4.7 ± 0.7 ms and 4.3 ± 0.7 ms at four and eight weeks, respectively. Concurrently, conduction velocity exhibited a progressive increase, with the median nerve improving from 48.5 ± 6.2 m/s to 51.1 ± 5.9 m/s and 54.3 ± 5.8 m/s, while the peroneal nerve increased from 40.2 ± 5.4 m/s to 43.0 ± 5.3 m/s and 46.1 ± 5.1 m/s over the same period (Table 4).

**Table 4:** Nerve conduction study parameters at baseline, four weeks, and eight weeks Data presented as mean ± SD. Repeated measures ANOVA applied; p < 0.05 is considered statistically significant.

Nerve	Parameter	Baseline (mean ± SD)	Four weeks (mean ± SD)	Eight weeks (Mean ± SD)	F-value	p-value
Median	Latency (ms)	4.2 ± 0.6	3.9 ± 0.5	3.5 ± 0.5	24.3	<0.001
Median	Conduction velocity (m/s)	48.5 ± 6.2	51.1 ± 5.9	54.3 ± 5.8	26.7	<0.001
Peroneal	Latency (ms)	5.1 ± 0.8	4.7 ± 0.7	4.3 ± 0.7	18.6	<0.001
Peroneal	Conduction velocity (m/s)	40.2 ± 5.4	43.0 ± 5.3	46.1 ± 5.1	22.9	<0.001

Adverse effects of duloxetine

Adverse effects were reported in 32 (28.3%) patients, with nausea (12, 10.6%), dizziness (8, 7.1%), and somnolence (7, 6.2%) being the most common (Table 5).

**Table 5:** Adverse effects of duloxetine Data presented as N (%) (descriptive statistics).

Adverse effect	N (%)
Nausea	12 (10.6%)
Dizziness	8 (7.1%)
Somnolence	7 (6.2%)
Dry mouth	5 (4.4%)
Sweating	3 (2.7%)
Total	32 (28.3%)

## Discussion

DPN is a significant microvascular complication of DM, affecting a large proportion of patients and contributing to chronic neuropathic pain, sensory deficits, and reduced quality of life [7]. This study aimed to evaluate the efficacy of duloxetine in the management of DN, focusing on its impact on pain severity, neuropathy symptoms, nerve conduction parameters, and adverse effects.

The study included 113 patients, with a mean age of 52.7 ± 8.1 years. The majority of patients were in the 35-55 age group (77, 68.1%), followed by 56-65 years (22, 19.5%). This age distribution aligns with previous studies, which reported that neuropathy prevalence increases with age due to cumulative metabolic stress and nerve damage [12]. The mean duration of diabetes was 6.3 ± 2.1 years, with 57.5% of patients having diabetes for ≤5 years, suggesting that neuropathy symptoms can develop in the early stages of diabetes, particularly in individuals with poor glycemic control [13]. The mean duration of neuropathy symptoms was 8.1 ± 3.5 months, with 58.4% of patients experiencing symptoms for more than six months, indicating chronic progression of the disease. Additionally, 50.4% of patients were overweight, and 18.6% were obese, reinforcing the established link between higher BMI and neuropathy severity [14,15].

Pain severity, as measured by the VAS, significantly decreased from 7.6 ± 1.1 at baseline to 4.3 ± 0.9 at four weeks and 2.5 ± 0.8 at eight weeks (p < 0.001). These findings align with previous randomized controlled trials, where duloxetine demonstrated superior pain relief compared to placebo, showing a significant reduction in 24-hour pain scores [16]. Duloxetine’s efficacy is attributed to its dual inhibition of serotonin and norepinephrine reuptake, which modulates descending pain pathways in the central nervous system [9]. Similar results were reported by Dash et al., where duloxetine therapy led to a substantial decrease in neuropathic pain over 12 weeks [17].

The MNSI score, which evaluates neuropathic symptom severity, improved significantly from 6.4 ± 1.3 at baseline to 5.2 ± 1.1 at four weeks and 4.1 ± 1.1 at eight weeks (p < 0.001). This reduction suggests a meaningful improvement in sensory function and neuropathic symptoms, consistent with findings by Sumedhan et al. [18], who noted that patients treated with duloxetine reported fewer pain episodes and greater symptom relief compared to placebo. Additionally, a placebo-controlled, multicentric study confirmed duloxetine’s superiority over placebo in achieving a 50% reduction in neuropathic pain [19].

NCS demonstrated significant improvements in key parameters of the median and peroneal nerves, supporting duloxetine’s potential neuroprotective effects. The median nerve latency improved from 4.2 ± 0.6 ms at baseline to 3.9 ± 0.5 ms at four weeks and 3.5 ± 0.5 ms at eight weeks (p < 0.001), while its conduction velocity increased from 48.5 ± 6.2 m/s to 51.1 ± 5.9 m/s at four weeks and 54.3 ± 5.8 m/s at eight weeks (p < 0.001). Similarly, the peroneal nerve latency improved from 5.1 ± 0.8 ms to 4.7 ± 0.7 ms at four weeks and 4.3 ± 0.7 ms at eight weeks (p < 0.001), with conduction velocity increasing from 40.2 ± 5.4 m/s to 43.0 ± 5.3 m/s at four weeks and 46.1 ± 5.1 m/s at eight weeks (p < 0.001). These results align with previous trials, where duloxetine demonstrated comparable nerve conduction improvements to pregabalin [20].

The safety and tolerability profile of duloxetine was favorable, with adverse effects reported in 32 (28.3%) patients. The most common side effects were nausea (12, 10.6%), dizziness (8, 7.1%), somnolence (7, 6.2%), dry mouth (5, 4.4%), and sweating (3, 2.7%). These findings are in accordance with previous clinical trials, where duloxetine had a higher incidence of nausea and somnolence compared to placebo, but most side effects were mild and transient [21]. Dash et al. reported higher discontinuation rates due to adverse effects, while our study found no patient discontinuation due to adverse effects, indicating good tolerability in this population [17]. However, in an open-label safety trial, discontinuation rates were significantly higher with duloxetine compared to pregabalin (p = 0.04), suggesting that individual tolerance may vary [18].

Glycemic parameters remained unchanged, with the mean HbA1c level at 7.8 ± 1.3%, FBG at 176.4 ± 21.5 mg/dL, and PPBS at 224.6 ± 28.1 mg/dL. These results are consistent with previous studies, which found no significant effect of duloxetine on glucose metabolism [22]. While Dash et al. reported a slight increase in HbA1c levels (p < 0.001), other trials concluded that duloxetine does not adversely impact glycemic control, reinforcing its safety in diabetic patients [17].

Despite the promising results, some limitations should be noted. The study duration was limited to eight weeks, and longer follow-up periods are needed to assess the sustainability of pain relief and neuropathic symptom improvement. Out of the initial 113 patients enrolled, 106 completed all scheduled follow-up assessments. Seven patients were lost to follow-up due to reasons including withdrawal of consent and non-compliance. Another limitation is that the MNSI and VAS assessments were not blinded and were conducted by the treating physicians, which may introduce potential observer bias in outcome evaluation. Additionally, while this study focused on monotherapy with duloxetine, future research should explore combination therapies with other agents such as pregabalin or gabapentin to determine the optimal treatment approach for DN. One key limitation of this study is the absence of a control or placebo group, which limits the ability to attribute the observed improvements solely to duloxetine and does not account for potential placebo effects or natural symptom variation.

## Conclusions

This study confirms that duloxetine is an effective and well-tolerated treatment for DN, leading to significant reductions in pain severity, improvements in neuropathic symptoms, and enhancements in nerve conduction parameters. Patients experienced notable relief from neuropathic pain, along with better sensory function and nerve conduction velocity, indicating potential neuroprotective effects. Duloxetine was generally well tolerated, with mild-to-moderate adverse effects, the most common being nausea, dizziness, and somnolence. Importantly, no significant impact on glycemic control was observed, reinforcing its safety profile for use in diabetic patients. Given the burden of DN and its negative impact on quality of life, these findings support the routine use of duloxetine as a first-line pharmacological option for managing neuropathic pain. However, longer follow-up studies and comparative trials with other treatments are needed to further establish its long-term efficacy and safety. Early intervention with effective therapies like duloxetine can help improve functional outcomes, reduce complications, and enhance overall patient well-being.
